# The Expression and Activity of Rhodanese, 3-Mercaptopyruvate Sulfurtransferase, Cystathionine γ-Lyase in the Most Frequently Chosen Cellular Research Models

**DOI:** 10.3390/biom11121859

**Published:** 2021-12-10

**Authors:** Marta Kaczor-Kamińska, Kamil Kaminski, Maria Wróbel

**Affiliations:** 1 Faculty of Medicine, Medical College, Chair of Medical Biochemistry, Jagiellonian University, Kopernika 7 St., 31-034 Krakow, Poland; mtk.wrobel@uj.edu.pl; 2 Faculty of Chemistry, Jagiellonian University, Gronostajowa 2 St., 30-387 Krakow, Poland; kaminski@chemia.uj.edu.pl

**Keywords:** cysteine, glutathione, polymorphism, sulfane sulfur, sulfurtransferases

## Abstract

This paper provides information concerning the activity and expression levels of three sulfurtransferases (STRs): rhodanese (TST, EC: 2.8.1.1), 3-mercaptopyruvate sulfurtransferase (MPST, EC: 2.8.1.2) and cystathionine γ-lyase (CTH, EC: 4.4.1.1) in various cell lines. Since very limited data are available in the scientific literature on this subject, the available data are included in this paper. These shortages often force the researchers to carry out their own screening tests that allow them to choose an appropriate model for their further studies. This work supplements the existing deficiencies in this area and presents the activity and expression of STRs in the eight most frequently chosen cell lines: the mouse mammary gland cell line (NMuNG, ATCC: CRL-1636), mouse mammary gland tumor (4T1, ATCC: CRL-2539), mouse fibroblast (MEF, ATCC: SCRC-1008), mouse melanoma (B16-F1, ATCC: CRL-6323), human colorectal adenocarcinoma (Caco-2, ATCC: HTB-37), human embryonic kidney (HEK-293, ATCC: CRL-1573), human osteosarcoma (MG-63, ATCC: CRL-1427) and rat myocardium (H9c2, ATCC: CRL-1446). Changes in STRs activity are directly related to the bioavailability of cysteine and the sulfane sulfur level, and thus the present authors also measured these parameters, as well as the level of glutathione (its reduced (GSH) and oxidized (GSSG) form) and the [GSH]/[GSSG] ratio that determines the antioxidant capacity of the cells. STRs demonstrate diverse functionality and clinical relevance; therefore, we also performed an analysis of genetic variation of STRs genes that revealed a large number of polymorphisms. Although STRs still provide challenges in several fields, responding to them could not only improve the understanding of various diseases, but may also provide a way to treat them.

## 1. Introduction

Rhodanase (thiosulfate sulfurtransferase, TST, EC: 2.8.1.1), 3-mercaptopyruvate sulfurtransferase (MPST, EC: 2.8.1.2) and cystathionine γ-lyase (CTH, EC: 4.4.1.1) belong to the enzyme superfamily known as sulfurtransferases (STRs). The enzymes are widespread in the world of prokaryotic and eukaryotic organisms, but, in recent years, they have rapidly become a subject of our growing interest; here, the trigger was the discovery that they were involved in the multi-enzyme pathway, which can generate hydrogen sulfide from cysteine, homocysteine and their disulfides [1,2,3,4,5,6].

TST and MPST have similar physicochemical and catalytical properties (Table 1) [7,8,9,10], because, as it was proved, they are evolutionary related enzymes [7,8,10,11,12]. The catalytic activity of the two enzymes is dependent on the cysteine residue in their active center [7,8,11,13,14,15]. The earliest known function of these enzymes was the direct participation in processes of cyanides detoxification. Nonetheless, CTH is also the enzyme that can directly take part in cyanide metabolism. However, CTH does not catalyze the oxidation of cyanide to SCN^-^ (as TST and MPST do), but produces sulfur-donor molecules for STRs [16]. Even though all three STRs participate in this process, they have a different substrate specificity, organ distribution and may have different roles in the regulation of cellular sulfur balance [17]. All of these enzymes are also involved in the L-cysteine metabolism pathways. CTH and MPST are mostly engaged in the processes of sulfane sulfur-containing compounds formation, while TST acting is rather restricted to the transfer of sulfane sulfur labile atoms (to know more about cysteine metabolism, please see [18,19,20]). The most important molecular functions of STRs, as well as the biological processes in which STRs are involved in, are collected in Table 1.

Genetic characterization of STRs is presented in Table 2. The table contains such data as genes localization and their length, number of exons, number of isoforms and their mRNA sequences and proteins length. Additionally, using the National Center of Biotechnology Information (NCBI) database, as well as the National Institute of Environmental Health Sciences (NIH) website, the present authors checked whether there were changes in the DNA of human STRs (single nucleotide polymorphism—SNP) that were found too often to be called a random mutation (differences in the population with a frequency above 1%).

**Figure 1:**
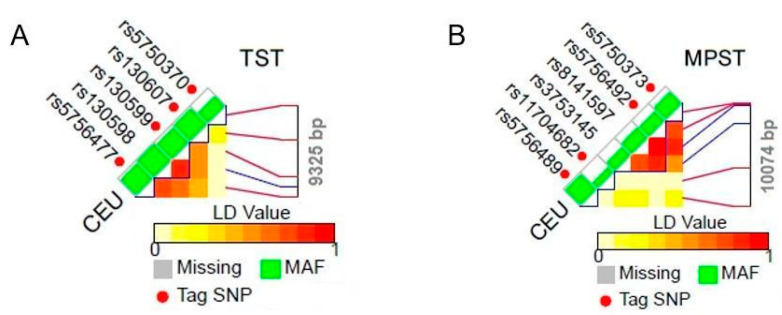
Chromosome position of LD patterns of tagSNP for TST (**A**) and MPST (**B**) gene of Utah Residents with Northern and Western European Ancestry (CEU) (according to the National Institute of Environmental Health Sciences). Abbreviations: MAF—minor allele frequency track.

**Figure 2:**
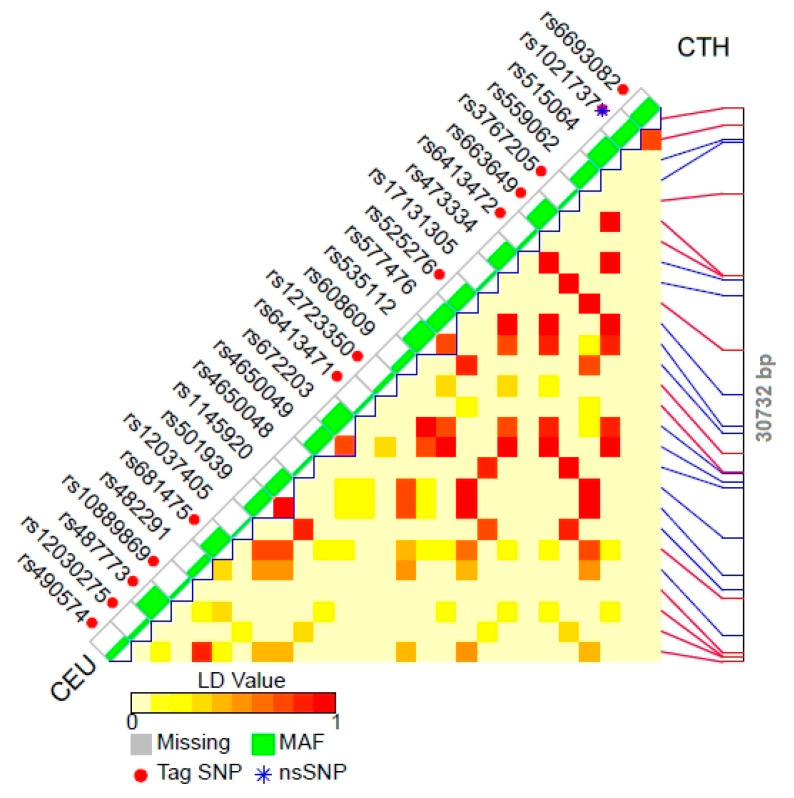
Chromosome position of LD patterns of tagSNP for CTH gene of Utah Residents with Northern and Western European Ancestry (CEU) (according to the National Institute of Environmental Health Sciences). Abbreviations: MAF—minor allele frequency track, nsSNP—non-synonymous single-nucleotide polymorphisms.

Based on the data collected in the 1000 Genomes Project, we found reference polymorphisms which could be inherited in conjunction with other genes (tag SNPs, Table 2). The chromosome position of linkage disequilibrium (LD) patterns of tagSNP for the appropriate gene of Utah Residents with Northern and Western European Ancestry (CEU) is shown in Figure 1A (TST), Figure 1B (MPST) and Figure 2 (CTH). Any mutation that occurs in genes encoding STRs may interfere with their proper functioning, mostly by affecting their expression and/or activity changes.

In various databases, e.g., the NCBI (browse by gene ID: 7263 ID for human TST, 4357 ID for human MPST, and 1491 ID for human CTH gene) or the Open Targets Platform (browse according to following targets for human genes: ENSG00000128311 for TST; ENSG00000128309 for MPST and ENSG00000116761 for CTH gene), there is some information available concerning STRs RNA and protein expression in various tissues, while the information addressing various cell lines, especially non-human cell lines, is still missing. These shortages often force the researchers to carry out their own screening tests in this direction, so that it will be possible to choose the appropriate research model for their further research. This work supplements the existing deficiencies in this area and presents the activity and expression of STRs in the most frequently chosen cellular research models. In addition, these data were supplemented with parameters such as sulfane sulfur level, cysteine level, reduced and oxidized glutathione levels, as well as the [GSH]/[GSSG] concentration ratio—the parameters that are very often determined along with the activity and expression of STRs.

The cell lines presented in this paper were selected based on responses to an anonymous questionnaire from the free online survey tool, Survey Monkey (www.surveymonkey.com, accessed on 22 February 2018). The questionnaire listed 80 cell lines diverse in species, tissue origin, and disease occurrence. The cell lines included in this study represent 10% of the most frequently chosen records from scientists employed by the Jagiellonian University, willing to participate in the survey. The present investigators also present data and references for five additional cell lines (the human neuroblastoma SH-SY5Y, human astrocytoma U373, human glioblastoma-astrocytoma U87MG, and two lines representing the murine cellular model of mucopolysaccharidosis, type IIIB: the WT (the control), and *Naglu*^−/−^ line (the line with mutation in gene encoding N-alpha-acetylglucosaminidase), since there are data from the scientific literature addressing this topic of interest.

## 2. Materials and Methods

### 2.1. Cell Culture

All experiments were performed using cell lines obtained from American Type Culture Collection (ATCC). We used eight cell lines: mouse mammary gland cell line (NMuMG, ATCC: CRL-1636), mouse mammary gland tumor (4T1—ATCC: CRL-2539)—this tumor mimics an animal stage IV human breast cancer—mouse fibroblast (MEF, ATCC: SCRC-1008), mouse melanoma (B16-F1, ATCC: CRL-6323), human colorectal adenocarcinoma (Caco-2, ATCC: HTB-37), human embryonic kidney (HEK-293, ATCC: CRL-1573), human osteosarcoma (MG-63, ATCC: CRL-1427) and rat myocardium (H9c2, ATCC: CRL-1446). All cell lines were grown in Dulbecco’s Modified Eagle’s Medium supplemented with 10% (*v*/*v*) fetal bovine serum and 100 U/mL penicillin and 100 μg/mL streptomycin. To make the complete growth medium of the NMuMG cells, the addition of insulin was necessary (final concentration of insulin in medium: 10 μg/mL). For experimental procedures cell lines were maintained in exponential growth and were refed 48 h prior to harvest. They were maintained at 37 °C, 5% CO_2_, 95% humidity.

### 2.2. Cell Homogenization

The pellets of cells (3.5–5 × 10^6^ cells) were suspended in 0.1 M phosphate buffer, pH 7.5, in the proportion 1 mln cells/0.04 mL of the buffer and sonicated 15 s at 4 °C (Bandelin Sonoplus GM70, Berlin, Germany). After centrifugation at 1600× *g*, at 4 °C for 10 min, the supernatant was used for determinations of protein content, sulfane sulfur levels and sulfurtransferases activity. For determinations of low-molecular-weight, sulfur-containing compounds using reverse-phase, high-performance liquid chromatography (RP-HPLC), the pellets were suspended in 250 μL 0.9% NaCl/1 mM BPDS/70% perchloric acid (PCA) and sonicated for 15 s at 4 °C (Bandelin Sonoplus GM70, Berlin, Germany). The sediment was separated by centrifugation at 1600× *g* for 10 min, and supernatant was saved at −80 °C until used for RP-HPLC analyses.

### 2.3. TST Activity Assay

The TST activity was assayed by the Sörbo’s method [21], following a procedure described by Wróbel et al. [22]. The incubation mixture contained: 200 μL of 0.125 M sodium thiosulfate, 100 μL 0.2 M potassium dihydrogen phosphate, 100 μL of homogenates, 100 μL 38% formaldehyde (only blank sample) and 100 μL of 0.25 M potassim cyanide. The mixture was incubated for 5 min at room temperature. Subsequently, 100 μL of 38% formaldehyde (only tested sample) and 500 μL of 0.2 M ferric nitrate reagent were added to all samples. The amount of thiocyanate formed during the reaction catalyzed by TST was measured colorimetrically at 460 nm. The enzyme units were defined as nmoles of SCN^−^, which formed during 1 min incubation per 1 mg protein.

### 2.4. MPST Activity Assay

The MPST activity was assayed according to the method of Valentine and Frankenfeld [23] with some modifications described by Wróbel et al. [22]. The incubation mixture contained: 250 μL of 0.12 M sodium phosphate buffer, pH 8.0; 50 μL of 0.5 M sodium sulfite; 50 μL of 0.15 M dithiothreitol; 50 μL of homogenates; 50 μL of distilled water; and 50 μL of 0.1 M 3-mercaptopyruvate acid sodium salt. The mixture was incubated for 15 min at 37 °C. After that, 250 μL of 1.2 M PCA was added to stop the reaction. Samples were centrifuged at 1600× *g* for 5 min. Then, 100 μL of supernatant was transferred to a prepared mixture consisting of 1200 μL of 0.12 M sodium phosphate buffer, pH 8.0; 100 μL of 0.1 M N-ethylmaleimide; and 50 μL of 7.5 mM β-Nicotinamide adenine dinucleotide reduced disodium salt hydrate (NADH). After equilibration at 37 °C, 2.5 μL of lactate dehydrogenase (LDH, 7 IU) was added, and the decrease in absorbance was measured at 340 nm. The difference between the initial value of absorbance (before LDH addition) and the lowest value (after LDH addition) corresponded to the amount of pyruvate formed in the reaction catalyzed by MPST. The MPST activity was expressed as nmoles of pyruvate produced during one-minute-incubation at 37 °C per 1 mg of protein.

### 2.5. CTH Activity Assay

The CTH activity was determined according to Matsuo and Greenberg’s method [24], which was modified by Wróbel et al. [22]. The incubation mixture contained: 25 μL of 1.3 mM pyridoxal phosphate; 25 μL of 0.02 mM EDTA; 250 μL of 45 mM cystathionine solution in 0.1 M phosphate buffer, pH 7.5 (2.5 mg of cystathionine per sample); and 75 μL of homogenate. A phosphate buffer of 0.1 M and pH 7.5, containing 0.05 mM 2-mercaptoethanol, was added to the samples to obtain a final mixture volume equal to 650 μL. After 30 min of incubation at 37 °C, the reaction was stopped by placing 125 μL of incubation mixture in 25 μL of 1.2 M PCA. Samples were centrifuged at 1600× *g* for 10 min. After that, 25 μL of supernatant was transferred to 625 μL of 0.194 mM NADH solution and kept at 37 °C. Control samples (without 24 mM cystathionine) were prepared in the same way as the examined samples. The measurement (absorbance at 340 nm) was conducted for 180 s; after 10 s, 25 μL of LDH (9.06 IU) was added. The difference between the initial value of absorbance (before LDH addition) and the lowest value (after LDH addition) corresponded to the amount of α-ketobutyrate formed in the reaction catalyzed by CTH. The enzyme activity was expressed as nmoles of α-ketobutyrate formed during a one-minute-incubation at 37 °C per 1 mg of protein.

### 2.6. The Sulfane Sulfur Level

Sulfane sulfur level was determined by the method of Wood [25] with some modifications. The method was based on cyanolysis reaction and colorimetric detection of ferric thiocyanate complex ion. Incubation mixtures in a final volume, 880 µL, contained: 20 μL 1 M ammonia solution, 20 μL homogenate, 740 μL distilled water and 100 μL 0.5 M potassium cyanide. The incubation was performed for 45 min at room temperature. After incubation, thiocyanate was estimated colorimetrically at 460 nm, after addition of 20 μL 38% formaldehyde and 40 μL 0.2 M ferric nitrate reagent. The level of sulfane sulfur was expressed as nmoles of SCN^-^ (thiocyanate) per 1 mg of protein.

### 2.7. The Protein Content Determination

Total protein content was determined by the method of Lowry et al. [26]. The crystalline bovine serum albumin was used as a standard.

### 2.8. Determination of Concentration of Low-Molecular-Weight Sulfur-Containing Compounds Using RP-HPLC

The levels of the reduced (GSH) and oxidized (GSSG) glutathione, cysteine and cystine in the incubation mixtures were determined using the RP-HPLC method of Dominic et al. [27] with modifications [28]. The samples were separated on a 4.6 mm × 250 mm Luna C18 (5 µm) column with a Phenomenex Security Guard column filled with the same packing material. The chromatographic system consisted of LC-10 Atvp Shimadzu Corp. pumps, four channel degassers, column oven, a Shimadzu SIL-10 Advp autosampler and a Shimadzu Corp. SIL-10 SPD-M10Avp-diode array detector; Lab Solution LC software was used to control system operation and facilitate data collection. The standard curves were generated in the supernatant obtained from cellular homogenates in the range from 13 to 75 nM of each compound per ml. All the standard curves generated for the analyte were linear in the investigated concertation range.

### 2.9. Total RNA Isolation

Total RNA was extracted from the cells using TRI reagent, according to the protocol provided by the manufacturer (Sigma-Aldrich, Darmstadt, Germany). The extracted RNA was suspended in ribonuclease free-water and quantified by measuring the absorbance at 260 nm. After the procedure, the purity of the obtained RNA was determined by the spectrophotometric analysis (A260 nm/A280 nm). The integrity of the achieved RNA was confirmed by the separation of the 28S and 18S rRNA bands in 2.0% agarose-gel electrophoresis. The RNA solutions were stored at −80 °C until further use.

### 2.10. Reverse Transcription of RNA

Total RNA from the cell samples was reverse-transcribed using a GoScript^TM^ Reverse Transcriptase Kit according to the manufacturer’s protocol (Promega, Madison, WI, USA). For a reverse transcription reaction, 3 µg of total RNA was mixed with 1 μL of Oligo (dT)_15_ primer (0.5 μg/reaction) and water pretreated with diethylpyrocarbonate (DEPC-H_2_O), and then incubated for 5 min at 70 °C. After preincubation, other components were added to this mixture: 4 μL of GoScript^TM^ 5× concentrated reaction buffer, 3 μL of MgCl_2_ (final concentration 1.5–5.0 mM), 1 μL of deoxyribonucleotide triphosphates (dNTPs, 10 mM), 1 μL of RNase inhibitor (20 U/μL), and 1 μL of GoScript^TM^ Reverse Transcriptase (160 U/μL) in a total volume of 20 μL. The mixture was first incubated for 5 min at 25 °C, then for 60 min at 42 °C, and, finally, for 15 min at 70 °C. If necessary, the solutions of complementary DNA (cDNA) were stored at −20 °C.

### 2.11. Polymerase Chain Reaction (PCR)

The expressions of the three genes (MPST, TST, CTH, GAPDH) were analyzed by PCR. As a reference (an internal standard), gene-encoding glyceraldehyde 3-phosphate dehydrogenase (GAPDH, gene expressed normally in cells) was used. The amplification of cDNA samples was performed in a 12.5 μL reaction volume containing 1 μL of synthesized cDNA, 10 μM of each of gene-specific primer pair, 2 U/μL Taq DNA polymerase in 10 mM buffer Tris-HCl at pH 8.8, and 10 mM of each dNTPs and DEPC-H_2_O. In each the case, a similar reaction was also performed in the mixture without DNA (the negative control) in order to confirm the specificity of the obtained reaction products. The temperature profile of PCR amplification for these genes, as well as gene-specific primer sequences, are collected in Table 3. The PCR reaction conditions for these four genes in three different species were established and optimized specifically to address the needs of the present study; they are published for the first time in this paper. All the amplification reactions were performed at least three times to ensure the accuracy of the results. All the PCR products were analyzed by electrophoresis on 2.0% agarose gel stained with ethidium bromide, directly visualized under UV light and photographed (ChemiDoc^TM^ MP Imaging system with Image Lab Software, version 6.0, Bio-Rad).

## 3. Results and Discussion

We performed an analysis of genetic variations of the TST-, MPST- and CTH-encoding genes (Table 2). Some information concerning CTH gene polymorphism was reported by us in 2014 [29]. In the present report, we updated these data and performed the same analysis for the other two STRs—TST and MPST. Based on the data presented in Table 2, the genes encoding STRs show a large number of polymorphisms. Two of these genes, rs1021737 and rs28941785 (Table 2), revealed in the coding region of CTH gene, cause a missense mutation by G > T and C > T/A transversion, respectively. Coding SNPs cause either a change in the amino acid sequence of the protein that is produced (non-synonymous SNPs change) or do not affect the protein sequence, because one codon is changed to another but still encodes the same amino acid (synonymous SNPs). However, the aforementioned replacements are pathogenic mutations that lead to cystathioninuria (OMIM 219500) [30]. These mutations may cause a change in the CTH expression level. Should these mutations cause the overexpression of the gene encoding CTH—the enzyme that catalyzes the conversion of cystathionine to cysteine, ammonia and 2-oxobutyrate—then, the cells trigger an increased production of hydrogen sulfide (H_2_S). H_2_S plays a role in protection of neurons against oxidative stress and stimulates an increase in γ-glutamylcysteine synthetase, and thereby an increase in the level of GSH. On the other hand, diminished CTH expression entails a decrease in the level of cysteine, glutathione (GSH), taurine and H_2_S in the cells [29,31]. The reduction/enhancement in gene expression and/or activity of H_2_S-generating enzymes (MPST, CTH) in the cells may causes a decrease/increase in H_2_S bioavailability and indicates the possible genetic background of H_2_S-geneting enzyme regulation [32]. Therefore, any mutation present in the genes encoding TST, MPST, or CTH disrupts the normal function of these enzymes. Even though most of the revealed polymorphisms (Table 2) of the tested STRs are not within the protein-coding regions, and can be classified as ‘likely benign’, or ‘benign’ (only a few of them are classified as of ‘uncertain significance’ according to NCBI), they still may affect gene splicing, transcription factor binding, messenger RNA degradation or the sequence of noncoding RNA. Mounting evidence suggests that noncoding SNPs, especially those that are localized in the vicinity of protein-coding genes (e.g., promoters, enhancers and 3′ termini sequence) play important roles in shaping chromatin structure, regulating gene expression, affecting mRNA structure and disease susceptibility [33] and/or increasing the risk of cancer [34]. Moreover, SNPs do not usually function individually, they rather work in coordination with other SNPs (polymorphisms inherited in conjunction) to cause a disease. Large-scale association studies were performed in an attempt to discover disease caused by SNPs within a varied population, but a large number of such conditions are still unknown. For example, there is an extremely rare (prevalence: <1/1,000,000) disease known as beta-mercaptolactate cysteine disulfiduria (MCDU, OMIM 249650), but to date there are no data available associated with any (one or more) of the revealed polymorphisms of the MPST gene (Table 2). MCDU is an autosomal, recessive, metabolic disorder resulting in a deficiency in MPST activity in erythrocytes.

**Figure 3:**
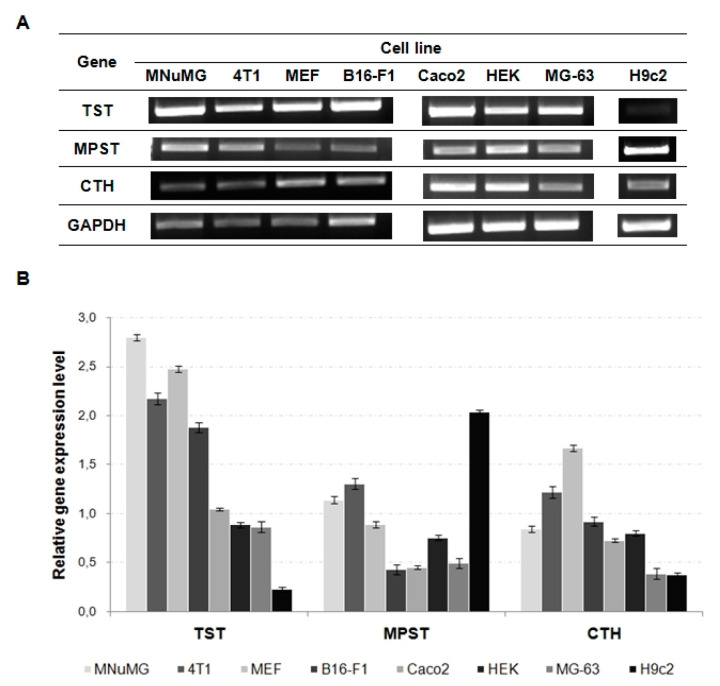
RT-PCR analysis. (**A**) Genes expression in the tested cell lines. (**B**) The relative expression level of TST, MPST and CTH in the tested cell lines. Densities of bands were normalized using the signal for the GAPDH gene. The results are representative and obtained from 2 to 3 tests.

In the scientific literature, the expression data of tested STRs are presented only in a few cell lines. Jurkowska et al. [35] showed the level of MPST and CTH expression in the human SH-SY5Y and U87MG cell lines and two melanomas: A375 and WM35. Kaczor-Kamińska et al. [36] extended this knowledge by adding data concerning the expression of TST, MPST and CTH in the WT and *Naglu*^−/−^ cell lines (the cellular model of mucopolysaccharidosis IIIB). Therefore, to expand this knowledge, the expression levels of the genes encoding TST, MPST and CTH were investigated in selected cell lines. The obtained data are presented in Figure 3.

The performed gene expression analysis confirmed that the expression of all three STRs took place in all investigated cell lines: the mouse mammary gland cells MNuMG, mouse mammary gland tumor 4T1, mouse fibroblast MEF, mouse melanoma B16-F1, human colorectal adenocarcinoma Caco-2, human embryonic kidney cells HEK, human osteosarcoma MG-63, and rat myocardium H9c2. Taking into consideration the species tested, the highest TST and CTH expression level was observed in the mouse cell lines, while the MPST expression was the highest in the rat H9c2 cell line (Figure 3). Based on the data collected in Figure 3, among the chosen cell lines from three different species, the highest level of TST expression was determined in the following cell lines: MNuMG (mouse), Caco-2 (human), the highest MPST expression was detected in the 4T1 (mouse) and HEK (human) cells, and the highest level of CTH expression was revealed in the MEF (mouse) and HEK (human) cells. We tested only one rat cell line; therefore, there is no possibility of conducting similar comparison in this case.

The STRs activity analysis confirmed the presence of the activity of TST, MPST and CTH in all the tested and some additional cell lines; the latter were added to Table 4 due to availability of these data in the scientific literature. Based on the data collected in Table 4, among all the investigated cell lines, the highest TST activity was observed in the Caco-2 cell line, the lowest—in the 4T1 and B16-F1 cell lines. The highest MPST activity was observed in the SH-SY5Y cell line [5], while the lowest—in the *Naglu*^−/−^ cell line—the line derived from mice with mutation B6.129S6-Naglu^tm1Efn^/J in the gene encoding the N-alpha-glucosaminidase [36], as well as in the B16-F1 cell line. The activity of CTH was low and mostly comparable in all the studied cell lines (Table 4). By comparing the specific activities values of the three STRs, it was observed that the activity of CTH was the lowest among the tested enzymes. In the investigated cell lines, in the vast majority of cases, MPST showed the highest activity among STRs (Table 4). There was only one exception: the Caco-2 cell line, where the highest STRs activity was shown by TST. Depending on the tested mouse or rat cell line, the activity of MPST was at least 2 times greater compared to the activity of TST determined in the same cell line (Table 4). Among the investigated human cell lines, the difference in MPST and TST activity either did not occur, as in the MG-63 cell line, or was marginal, as in the HEK cell line. However, this observation suggests that, in almost all the tested cells, MPST can be the principal enzyme involved in sulfane sulfur-containing compounds formation and together with CTH, it participates in direct and/or indirect reactions leading to formation of H_2_S from cysteine, homocysteine and their disulfides [2].

Taking into consideration the level of sulfane sulfur-containing compounds in the tested cell lines (Table 5), we observed that their value was in the range: 41 nmole/mg protein (the SH-SY5Y cells); 190 nmole/mg protein (the MNuMG cells). In cells, sulfane sulfur-containing compounds are produced in the non-oxidative metabolism of cysteine [36,39]. In all the tested lines, the obtained values of the cysteine level were low and equaled on average ca. 0.5 nmole/mg protein (Table 5). The highest value of cysteine level was observed in the *Naglu*^−/−^ cells (2 nmole/mg protein), and this is likely a biochemical cellular response to the mutation existing in this line (the mutation likely causes an increase in the sulfate levels originating from undigested disaccharide—heparan sulfate) [36,40]. L-cysteine is also a direct substrate for GSH formation, and the results in Table 5 provide information about the glutathione level in the tested cell lines. The obtained results demonstrated that the reduced glutathione (GSH) level in the cells was at least one order of magnitude higher than the determined cysteine levels. There is only one exception—the *Naglu*^−/−^ cell line (Table 5). Furthermore, based on the analysis by the Mann–Whitney U test (*p* < 0.05), it can be concluded that, in the MG-63, SH-SY5Y and H9c2 cells, the low level of GSH and sulfane sulfur-containing compounds (Table 5) corresponded with a higher activity of CTH and MPST (in the SH-SY5Y cells) (Table 4) and lower CTH expression level (Figure 3). In contrast, in the MNuMG, HEK and B16-F1 cell lines, the high (the MNuMG line) or moderate (the HEK and B16-F1 lines) levels of GSH and sulfane sulfur (Table 5) were correlated with a lower CTH activity (Table 4) and the expression (Figure 3). The low activity of MPST in the 4T1, MEF, B16-F1, *Naglu*^−/−^ and Caco-2 cells and high activity of TST (the Caco-2 cells) (Table 4) were also related with the low level of sulfane sulfur-containing compounds and moderate or high (the Caco-2 line) level of GSH in these lines (Table 5). The most complex relationships occurred when GSH and sulfane sulfur levels were analyzed in relation to TST activity and expression. Thus, the low TST activity, but the high TST expression (Figure 3) determined in the 4T1, MEF, B16-F1 and *Naglu*^−/−^ cell lines (Table 4) was correlated with moderate sulfane sulfur and GSH levels in the lines (Table 5). Meanwhile, in the MNuMG cell line, the expression level of TST was high, TST activity was moderate, but the levels of GSH and sulfane sulfur were the highest among all investigated cell lines. However, in all the studied cell lines, the [GSH]/[GSSG] ratio determines the antioxidant capacity of the cells. Therefore, the data presented in Table 5 indicate the weakest antioxidant potential, among the tested cell lines, in the Caco-2 cells, while the strongest antioxidant potential was indicated in the B16-F1 cells.

The scientific literature presents a hypothesis postulating a decrease in STRs activity and the level of sulfane sulfur-containing compounds in neoplastic cell lines [35,37,42]. The mouse MNuMG line is very often used as a reference in studies incorporating the 4T1 cell line. Therefore, our results obtained for these two cell lines (Table 4 and Table 5) suggest that the aforementioned assumption appears to be true. The activity of TST and MPST determined in the 4T1 line is at least two times lower compared to the activity of these enzymes determined in the MNuMG cell line (Table 4). Similarly, the level of sulfane sulfur-containing compounds in the 4T1 line is one-half of the value determined in its reference line MNuMG (Table 5). It is difficult to analyze the predominantly low values of STRs activity determined in other neoplastic cell lines due to the lack of corresponding controls in the selected cell lines. However, we can observe that STRs activity (TST, MPST, CTH) (Table 4), as well as sulfane sulfur levels (Table 5) were also decreased in the cellular model of mucopolysaccharidosis, type IIIB [36]. Kruithof et al. [43] showed that TST deficiency was strongly related to the pathophysiology of metabolic diseases, including diabetes and obesity. There is some evidence of STRs activity playing a role in several other diseases, such as Lebers Hereditary Optic Disease (LHOD) (TST) [44,45], human gliomas (TST, MPST, CTH) [46] and in hemodialysis patients (TST) [47]. Therefore, these enzymes provide challenges in several fields and responding to them could not only improve the understanding of various diseases but may also provide a way to treat them.

## 4. Conclusions

Our studies of the STRs activity and expression levels and directly related parameters (the levels of cysteine, sulfane sulfur, oxidized and reduced glutathione, and the [GSH]/[GSSG] ratio) provide researchers with a choice of cellular research models without the need to conduct their own large-scale screening studies. The presented investigation results confirm the expression and activity of STRs in all the tested cell lines and demonstrate differences in the activity and the level of STRs mRNA depending on the species and origin the cells. Taking into consideration the species tested, the greatest TST and CTH expression was observed in the mouse cell lines, while the MPST expression was the highest in the rat H9c2 cell line (Figure 3). Among the chosen cell lines, the present authors demonstrated the highest level of STRs expression in the following cell lines: TST—the MNuMG cells, MPST—the H9c2 cells, and CTH—the MEF cells. We also confirmed that, irrespective of the cell species and origin, MPST activity was the highest (there was only one exception—the Caco2 cell line) and CTH activity was the lowest (Table 4), which indicated that sulfane sulfur-containing compounds were mainly formed in reactions catalyzed by MPST. The sulfane sulfur-containing compounds level in the tested cell lines was in the range: 41 (the SH-SY5Y cells) to 190 (the MNuMG cells) nmole/mg protein (Table 5). We also confirmed that the STRs activity, as well as the level of sulfane sulfur in the abnormal cell lines (neoplastic or otherwise pathologically altered) was lower compared to their reference lines (Table 4 and Table 5). The present authors also showed that the glutathione level in all the tested cell lines was at least one order of magnitude higher than the determined cysteine levels, as well as that the Caco-2 cells, which had the weakest antioxidant cells capacity, while the B16-F1 cells had the strongest (Table 5). Moreover, using information found in various databases, we collected and compiled the most important information concerning STRs (location, function, and genetic characteristics). These data allowed for conducting an analysis of the polymorphisms that occur in the genes encoding STRs. Therefore, for the first time, in this work, we also present a summary of such polymorphisms along with additional data on their positions on the chromosomes and information regarding which of these polymorphisms are inherited in conjugation with other polymorphisms, including those that are responsible for pathogenic states. These data and the data obtained confirm that STRs, as well as sulfane sulfur-containing compounds, play important roles in the regulation of many cellular functions (Table 1) and cellular homeostasis maintenance in various cell lines.

## Figures and Tables

**Table 1:** Sulfurtransferases localization and function. The search was performed in the Protein Data Bank (PDB) and in the Human Protein Atlas (HPA).

	TST	MPST	CTH
Subcellular (mammals) localization	mitochondrion	cytosol mitochondrion synaptosome	cytoplasm
Amino acids in the catalytic center of the enzyme	Arg-186, Cys-248, Lys-249	Arg-187; Arg-196; Cys-247; Ser-249	Ser-89; Gly-90; Leu-91; Met-110; Tyr-114; Asn-118; Thr-211; Lys-212; Met-214
Molecular function	5S rRNA binding transfers sulfur atom from thiosulfate to nucleophilic acceptors such as cyanide, forming persulfide intermediates	transfers sulfur atom from 3-mercaptopyruvate (or thiosulfate—week rhodanese activity) to thiol (RSH), persulfide (RSSH), cyanide, thioredoxin (Trx)	Cystathionine γ-lyase activity Carbon-sulfur lyase activity L-cysteine desulfhydrase activity L-cystine L-cysteine-lyase (deaminating) Pyridoxal phosphate binding (a cofactor)
Biological process participation	cyanate catabolic process thiosulfate generation—sulfane sulfur transfer to sulfite formation of iron-sulfur clusters in proteins antioxidative processes—thioredoxin oxidase activity 5S rRNA import into mitochondrion	hydrogen sulfide biosynthesis sulfane sulfur generation (thiosulfate) cyanate catabolic process antioxidative processes—thioredoxin peroxidase activity	hydrogen sulfide biosynthesis L-cysteine desulfhydrase activity cysteine biosynthesis protein sulfhydration transsulfuration pathway

**Table 2:** Genetic characterization of sulfurtransferases. The search was performed in the National Center of Biotechnology Information (NCBI). The tagSNPs names and polymorphisms inherited in conjunction were retrieved from the National Institute of Environmental Health Sciences website (https://www.niehs.nih.gov/ (accessed on 31 October 2021)) with default parameters and verified using dbSNP mode in the NCBI database.

Homo Sapiens	TST	MPST	CTH
Gene location	22q12.3	22q13.3	1p31.1
Gene length	9325 bp (NC_000022.11)	10074 bp (NC_000022.11)	30732 bp (NC_000001.11)
Number of exons	4	6	13
Number of isoforms (length of their mRNA and protein sequence encoded by them)	2 isoforms: Variant 1 (encodes longer transcript): mRNA: 1125 bp (NM_003312.6) protein: 297 aa (NP_003303.2) Variant 2 (difference in the 5’ UTR region): mRNA: 1111 bp (NM_001270483.1) protein: 297 aa (NP_001257412.1)	3 isoforms (5 different transcript variants): Variant 1 (1st isoform): mRNA: 1350 bp (NM_021126.8) protein: 317 aa (NP_066949.2) Variant 2 (2nd isoform): mRNA: 1252 bp (NM_001013436.4) protein: 297 aa (NP_001013454.1) Variant 3 (2nd isoform): mRNA: 1428 bp (NM_001130517.4) protein: 297 aa (NP_001123989.1) Variant 4 (2nd isoform): mRNA: 1448 bp (NM_001369904.2) protein: 297 aa (NP_001356833.1) Variant 5 (3rd isoform): mRNA: 1369 bp (NM_001369905.2) protein: 237 aa (NP_001356834.1)	3 isoforms: Variant 1 (encodes the longest transcript): mRNA: 2090 bp (NM_001902.6) protein: 405 aa (NP_001893.2) Variant 2 (lacks an in-frame exon in the coding region): mRNA: 2008 bp (NM_153742.4) protein: 361 aa (NP_714964.2) Variant 3 (lacks an in-frame exon in the coding region): mRNA: 2044 bp (NM_001190463.1) protein: 373 aa (NP_001177392.1)
Amount of single nucleotide polymorphism in whole gene and its coding regions (cSNP)	In whole gene: 2896 SNP Variant 1: 342 (cSNP) Variant 2: 342 (cSNP)	In whole gene: 3138 SNP Variant 1: 412 (cSNP) Variant 2: 386 (cSNP) Variant 3: 386 (cSNP) Variant 4: no data available Variant 5: no data available	In whole gene: 6835 SNP Variant 1: 389 (cSNP) Variant 2: 338 (cSNP) Variant 3: 363 (cSNP)
Type of mutation in the longest isoform coding region:	Isoform 1: 7 frame shift mutation 130 synonymous mutations 261 missense mutations	Isoform 1: 18 frame shift mutations 145 synonymous mutations 293 missense mutations 2 insertions 3 deletions	Isoform 1: 20 frame shift mutations 100 synonymous mutations 300 missense mutations
Tag SNPs (localization and effect)	**rs130599** (intron, T > C transversion); **rs5750370** (intron, G > A transversion); **rs5756477** (intron, T > A/C transversion); **rs130607** (intron, T > A/C/G transversion);	**rs5756492** (intron, G > A transversion); **rs5750373** (intron, G > A transversion); **rs5756489** (intron, T > A/C/G transversion); **rs11704682** (intron, C > A/G transversion);	**rs663649** (intron, G > T transversion); **rs525276** (intron, G > C/T transversion); **rs3767205** (intron, G > A/C/T transversion); **rs490574** (intron, T > A/G transversion); **rs6413471** (intron, A > C transversion); **rs487773** (intron, G > A/T transversion); **rs10889869** (intron, G > A transversion); **rs12030275** (intron, T > A/C transversion); **rs681475** (intron, T > C transversion); **rs12723350** (intron, T > C transversion); **rs1021737** (exon, G > T transversion, pathogenic mutation); **rs6693082** (3’ near gene, T > C/G transversion); **rs6413472** (intron, C > T transversion)
Polymorphisms inherited in conjunction	**rs130599** is inherited with: rs130598	**rs5756492** is inherited with: rs3753145, rs8141597; **rs5750373** is inherited with: rs8141597	**rs663649** is inherited with: rs4650049, rs473334, rs515064, rs535112, rs577476, rs672203; **rs525276** is inherited with: rs1145920, rs501939, rs559062, rs608609; **rs3767205** is inherited with: rs12037405, rs17131305, rs4650048; **rs490574** is inherited with: 482291
Polymorphisms responsible for pathogenic state	not revealed	not revealed	**rs1021737** (exon, G > T transversion, missense mutation) **rs28941785** (exon, C > T/A transversion) missense mutation) **rs28941786** (exon, C > G transversion, missense mutation or C > T transversion, nonsense mutation) **rs773107808** (exon, C > T transversion, nonsense mutation)

**Table 3:** Primer sequences and PCR conditions.

Gene	Primer (5′ → 3′)	Product Size (bp)	PCR Conditions					
			Predenaturation	Denaturation	Annealing	Elongation	Extension	Cycle
Mouse cell lines								
TST	F: AACCTGGGCATAAGCAACGA R: GGTCCACCTTCTTGTCCTGG	460	94 °C, 5 min	94 °C, 30 s	58 °C, 30 s	72 °C, 2 min	72 °C, 8 min	29
MPST	F: AACCTGGGCATAAGCAACGA R: GGTCCACCTTCTTGTCCTGG	420			62 °C, 30 s			30
CTH	F: CAGCAAGACCCGATGCAAAG R: CAAAGCAACACCTCGCACTC	304			60 °C, 30 s			
GAPDH	F: GCTCCAGCTTAGGTTCATCAG R: TTTGGCTCCACCCTTCAAGT	404			59 °C, 30 s			28
Human cell lines								
TST	F: GTCCGGGGCGAGTGACA R: GGCCAAACACACGGAACAT	393	94°C, 5 min	94°C, 30 s	59 °C, 30 s	72 °C, 2 min	72 °C, 8 min	30
MPST	F: CCAGGTACCGTGAACATCCC R: ATGTACCACTCCACCCAGGA	227			56 °C, 30 s			29
CTH	F: AATCGCTGTGTGCCGCCTTA R: TGAACGTGGTGGACAGTGAG	301			60 °C, 30 s		72 °C, 10 min	31
GAPDH	F: GCTCTCTGCTCCTCCTGTTC R: TTCCCGTTCTCAGCCTTGAC	273					72 °C, 8 min	30
Rat cell line								
TST	F: ACATCCGTGGCTCTGTCAAC R: TCGTAAACAGCCACATCGGG	208	94 °C, 5 min	94 °C, 30 s	62 °C, 30 s	72 °C, 2 min	72 °C, 10 min	30
MPST	F: GTATCTGCTCAGTGGGTGGC R: CAGGGATGTGTCCAGGTTCG	589						
CTH	F: CCGACGAGGAATTGCTTGGA R: ACATCACTGTGGCCGTTCAT	464			60 °C, 30 s			28
GAPDH	F: AGTGCCAGCCTCGTCTCATA R: GATGGTGATGGGTTTCCCGT	248						30

All the mRNA sequences of the tested genes were obtained from the National Center for Biotechnology Information (NCBI), and all of the primer sequences were synthesized by the DNA Sequencing and Synthesis Service—Institute of Biochemistry and Biophysics Polish Academy of Sciences (IBB PAN) in Warsaw, Poland.

**Table 4:** Sulfurtransferases activity in the selected cell lines.

Cell Line	TST	MPST	CTH	References
	nmole/mg·min^−1^			
Mouse cell lines				
MNuMG	61 ± 16	145 ± 34	0.6 ± 0.2	
4T1	14 ± 5	84 ± 19	1.6 ± 0.6	
MEF	31 ± 7	93 ± 14	0.8 ± 0.4	
B16-F1	25 ± 7	61 ± 14	0.4 ± 0.03	
WT	45 ± 5	81 ± 16	1.6 ± 0.5	[36]
Naglu^−/−^	26 ± 4	49 ± 15	0.9 ± 0.2	
Human cell lines				
Caco-2	502 ± 21	89 ± 44	1.8 ± 0.6	
HEK	182 ± 13	227 ± 54	0.8 ± 0.4	
MG-63	76 ± 18	77 ± 17	5.5 ± 0.7	
U373	70 ± 12	164 ± 20	no data	[37]
SH-SY5Y	67 ± 7	674 ± 93	5.2 ± 1.5	[5,38]
U87MG	26 ± 1	196 ± 23	3.3 ± 0.4	
Rat cell line				
H9c2	59 ± 16	110 ± 19	2.7 ± 0.8	

The values represent the arithmetic means ± SD of three independent experiments, with each determination consisting of 4–15 assays.

**Table 5:** Sulfane sulfur and low-molecular-weight thiol levels in the selected cell lines.

Cell Line	Sulfane Sulfur	Cysteine	GSH	GSSG	[GSH]/ [GSSG]	References
	nmole/mg Protein					
Mouse cell lines						
MNuMG	190 ± 42	<LOQ	64 ± 2	3 ± 0.2	21 ± 2	
4T1	83 ± 9	0.3 ± 0.2	17 ± 3	1 ± 0.1	12 ± 2	
MEF	94 ± 17	0.2 ± 0.1	28 ± 6	2 ± 0.5	15 ± 5	
B16-F1	124 ± 5	1 ± 0.1	37 ± 1	1 ± 0.3	33 ± 9	
WT	176 ± 23	2 ± 0.04	38 ± 4	4 ± 0.4	11 ± 0.04	[36]
*Naglu* ^−/−^	126 ± 18	2 ± 0.01	7 ± 1	1 ± 0.1	10 ± 2	
Human cell lines						
Caco-2	93 ± 12	0.5 ± 0.1	60 ± 7	7 ± 1	8 ± 1	
HEK	140 ± 7	0.2 ^a^	35 ± 2	3 ± 0.2	11 ± 1	
MG-63	65 ± 12	0.2 ^a^	15 ± 2	1 ± 0.1	20 ± 4	
U373	147 ± 23	no data	13 ± 2 ^#^	no data	no data	[37]
SH-SY5Y	41 ± 15	no data	1.8	no data	no data	[5]
U87MG	139 ± 47	no data	2.4	no data	no data	
Rat cell line						
H9c2	69 ± 17	0.5 ^a^	14 ± 2	0.3 ^a^	NA	

<LOQ—lower than the limit of quantification of the method; NA—not applied; ^#^—the GSH level determined by a modification of the enzymatic assay originally described by Tietze (1969) [37,41]; ^a^—the standard deviation was not calculated because of a low number of results. The data presented are the arithmetic mean of 3–4 determinations. The limit of detection for glutathione (GSH) in the RP-HPLC method is equal to 0.01 (nM·mL^−1^) and for oxidized form of glutathione (GSSG)—to 0.1 (nM·mL^−1^). The limit of quantification for GSH is 0.1 (nM·mL^−1^) and GSSG: 1 (nM·mL^−1^) [27]. The limit of detection for cysteine (CSH) was defined in the RP-HPLC method and is equal to 0.01 (nM·mL^−1^) and for cystine (CSSC)—to 0.1 (nM·mL^−1^). The limit of quantification for CSH: 0.1 (nM·mL^−1^) and the CSSC: 1 (nM·mL^−1^) [27].

## Data Availability

Not applicable.
